# (6bS*,14R*,14aR*)-Methyl 14-(4-methyl­phen­yl)-7-oxo-6b,6c,7,12b,14,14a-hexa­hydro-1*H*-pyrano[3,2-*c*:5,4-*c*′]dichromene-14a-carboxyl­ate

**DOI:** 10.1107/S1600536813001244

**Published:** 2013-01-19

**Authors:** R. Ponnusamy, V. Sabari, G. Sivakumar, M. Bakthadoss, S. Aravindhan

**Affiliations:** a Department of Computer Science & Engineering, Madha Engineering College, Kundrathur, Chennai 600 069, India; b Department of Physics, Presidency College, Chennai 600 005, India; c Department of Organic Chemistry, University of Madras, Chennai 600 025, India

## Abstract

In the title compound, C_28_H_22_O_6_, the chromeno ring system is almost planar, with a dihedral angle between the mean planes of the pyran and benzene rings of 1.87 (8)°. The pyran ring bearing the methyl­phenyl substituent has a half-chair conformation while the other pyran ring has an envelope conformation with the tetra­substituted C atom as the flap. The benzene ring of the chromeno ring system is inclined to the benzene ring fused to the latter pyran ring by 74.66 (9)°. These aromatic rings are inclined to the 4-methyl­phenyl ring by 52.67 (9) and 66.63 (10)°, respectively. In the crystal, mol­ecules are linked *via* C—H⋯O hydrogen bonds, forming a two-dimensional network parallel to the *bc* plane.

## Related literature

For the biological importance of 4*H*-chromene derivatives, see: Cai *et al.* (2006 ▶); Cai (2007 ▶, 2008 ▶); Gabor (1988 ▶); Brooks (1998 ▶); Valenti *et al.* (1993 ▶); Hyana & Saimoto (1987 ▶); Tang *et al.* (2007 ▶).
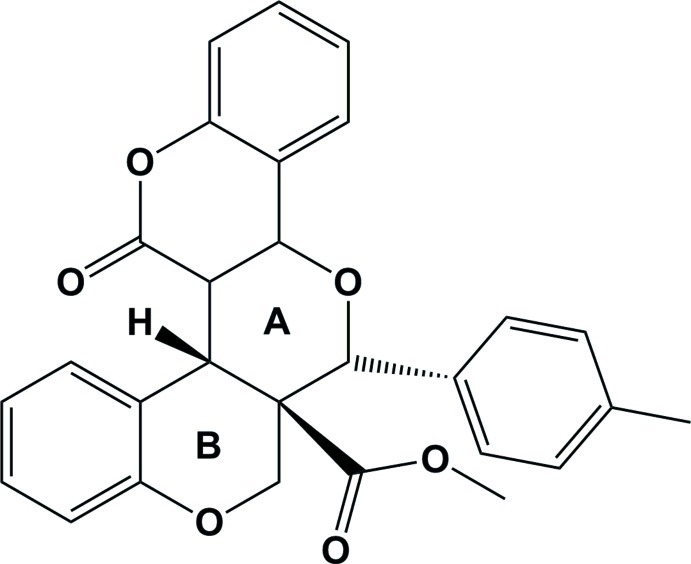


## Experimental

### 

#### Crystal data

C_28_H_22_O_6_
*M* *_r_* = 454.46
Monoclinic, 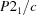

*a* = 9.526 (5) Å
*b* = 10.711 (5) Å
*c* = 21.975 (5) Å
β = 97.397 (5)°
*V* = 2223.5 (16) Å^3^
*Z* = 4
Mo *K*α radiation
μ = 0.10 mm^−1^
*T* = 293 K
0.32 × 0.20 × 0.10 mm

#### Data collection

Bruker APEXII CCD area-detector diffractometer
Absorption correction: multi-scan (*SADABS*; Bruker, 2008 ▶) *T*_min_ = 0.972, *T*_max_ = 0.992
22624 measured reflections
4741 independent reflections
3251 reflections with *I* > 2σ(*I*)
*R*_int_ = 0.028

#### Refinement

*R*[*F* ^2^ > 2σ(*F* ^2^)] = 0.045
*wR*(*F* ^2^) = 0.133
*S* = 1.01
4741 reflections
324 parameters
H atoms treated by a mixture of independent and constrained refinement
Δρ_max_ = 0.29 e Å^−3^
Δρ_min_ = −0.16 e Å^−3^

### 

Data collection: *APEX2* (Bruker, 2008 ▶); cell refinement: *SAINT* (Bruker, 2008 ▶); data reduction: *SAINT*; program(s) used to solve structure: *SHELXS97* (Sheldrick, 2008 ▶); program(s) used to refine structure: *SHELXL97* (Sheldrick, 2008 ▶); molecular graphics: *ORTEP-3* (Farrugia, 2012 ▶); software used to prepare material for publication: *SHELXL97* and *PLATON* (Spek, 2009 ▶).

## Supplementary Material

Crystal structure: contains datablock(s) I, global. DOI: 10.1107/S1600536813001244/su2541sup1.cif

Structure factors: contains datablock(s) I. DOI: 10.1107/S1600536813001244/su2541Isup2.hkl

Supplementary material file. DOI: 10.1107/S1600536813001244/su2541Isup3.cml

Additional supplementary materials:  crystallographic information; 3D view; checkCIF report

## Figures and Tables

**Table 1:** Hydrogen-bond geometry (Å, °)

*D*—H⋯*A*	*D*—H	H⋯*A*	*D*⋯*A*	*D*—H⋯*A*
C4—H4⋯O5^i^	0.93	2.35	3.209 (2)	153
C21—H21*A*⋯O5^ii^	0.96	2.43	3.285 (3)	148

Symmetry codes: (i) 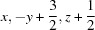; (ii) 
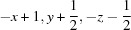
.

**Fig. 1.**
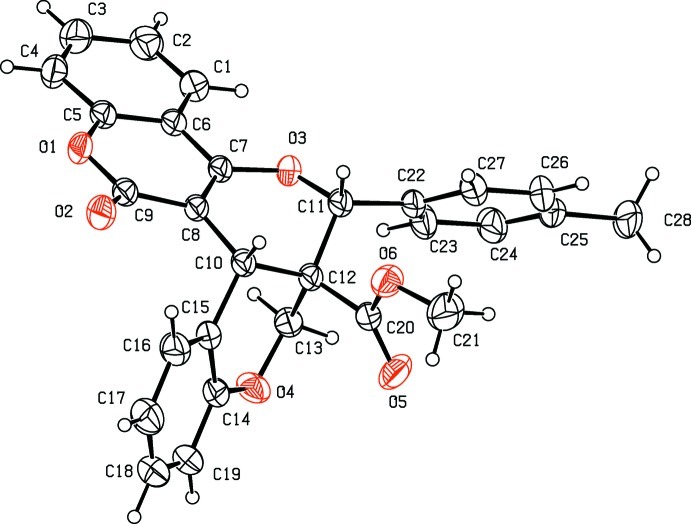
The molecular structure of the title molecule, with the atom numbering. The displacement ellipsoids are drawn at the 30% probability level.

**Fig. 2.**
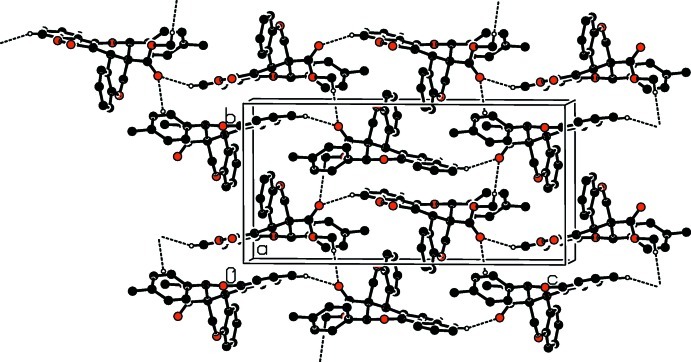
A view along the a axis of the crystal packing of the title compound. The C-H···O hydrogen bonds are shown as dashed lines [see Table 1 for details; H atoms not involved in these interactions have been omitted for clarity].

## supplementary crystallographic information

### Comment

4*H*-Chromenes are biologically important compounds used as synthetic ligands for drug design and discovery processes. They exhibit numerous biological and pharmacological properties such as anti-viral, anti-fungal, anti-inflammatory, anti-diabetic, cardionthonic, anti-anaphylactic and anti-cancer activity (Cai *et al.*, 2006; Cai, 2007,2008; Gabor, 1988; Brooks, 1998; Valenti *et al.*, 1993; Hyana & Saimoto, 1987; Tang *et al.*, 2007). In view of the growing medicinal importance of 4*H*-chromene derivatives, the title compound was synthesized and we describe herein its crystal structure.

The molecular structure of the title molecule is illustrated in Fig. 1. The mean plane of pyran ring A and the benzene ring (C1—C6) of the chromeno system are inclined to one another by 1.87 (8) °. Atom O2 deviates by 0.1987 (13) Å from the mean plane of the chromeno ring system (O1,C1—C9). Pyran ring A (O3/C7/C8/C10-C12) has a half-chair conformation while pyran ring B (O4/C10/C12-C15) has an envelope conformation with the tetra-substituted C atom, C12, as the flap. Benzene ring (C1—C6) of the chromeno ring system is inclined to the benzene ring (C14-C19) fused to pyran ring B by 74.66 (9) °. The 4-methylphenyl ring (C22-C27) is inclined to benzene rings (C1-C6) and (C14-C19) by 52.67 (9) and 66.63 (10) °, respectively.

In the crystal, molecules are linked via C—H···O hydrogen bonds forming a two-dimensional network lying parallel to the bc plane (Table 1 and Fig. 2).

### Experimental

A mixture of (*E*)-methyl 2-((2-formylphenoxy)methyl)-3-(4-methylphenyl) acrylate (0.308 g, 1 mmol) and 4-hydroxy-2H-chromen-2-one (0.162 g, 1 mmol) was placed in a round bottom flask and melted at 453 K for 1 h. After completion of the reaction as indicated by TLC, the crude product was washed with 5 ml of ethylacetate:hexane mixture (1:49 ratio) which successfully provided the title compound as a colourless solid in 97% yield. Diffraction quality crystals were obtained by slow evaporation of a solution in ethyl acetate.

### Refinement

The methine and methylene H atoms were located in a difference Fourier map and freely refined. The aromatic and methyl H atoms were included in calculated positions and treated as riding atoms: C—H = 0.93 Å (aromatic) and 0.96 Å (methyl) Å, with *U*_iso_(H) = 1.5U_eq_(C-methyl) and = 1.2U_eq_(C) for other H atoms.

### Crystal data

**Table:**

C_28_H_22_O_6_	*F*(000) = 952
*M**_r_* = 454.46	*D*_x_ = 1.358 Mg m^−^^3^
Monoclinic, *P*2_1_/*c*	Mo *K*α radiation, λ = 0.71073 Å
Hall symbol: -P 2ybc	Cell parameters from 5710 reflections
*a* = 9.526 (5) Å	θ = 1.8–28.5°
*b* = 10.711 (5) Å	µ = 0.10 mm^−^^1^
*c* = 21.975 (5) Å	*T* = 293 K
β = 97.397 (5)°	Monoclinic, colourless
*V* = 2223.5 (16) Å^3^	0.32 × 0.20 × 0.10 mm
*Z* = 4	

### Data collection

**Table:**

Bruker APEXII CCD area-detector diffractometer	4741 independent reflections
Radiation source: fine-focus sealed tube	3251 reflections with *I* > 2σ(*I*)
Graphite monochromator	*R*_int_ = 0.028
ω and φ scans	θ_max_ = 26.8°, θ_min_ = 2.1°
Absorption correction: multi-scan (*SADABS*; Bruker, 2008)	*h* = −12→11
*T*_min_ = 0.972, *T*_max_ = 0.992	*k* = −13→13
22624 measured reflections	*l* = −26→27

### Refinement

**Table:**

Refinement on *F*^2^	Secondary atom site location: difference Fourier map
Least-squares matrix: full	Hydrogen site location: inferred from neighbouring sites
*R*[*F*^2^ > 2σ(*F*^2^)] = 0.045	H atoms treated by a mixture of independent and constrained refinement
*wR*(*F*^2^) = 0.133	*w* = 1/[σ^2^(*F*_o_^2^) + (0.0626*P*)^2^ + 0.4826*P*] where *P* = (*F*_o_^2^ + 2*F*_c_^2^)/3
*S* = 1.01	(Δ/σ)_max_ < 0.001
4741 reflections	Δρ_max_ = 0.29 e Å^−^^3^
324 parameters	Δρ_min_ = −0.16 e Å^−^^3^
0 restraints	Extinction correction: *SHELXL97* (Sheldrick, 2008), Fc^*^=kFc[1+0.001xFc^2^λ^3^/sin(2θ)]^-1/4^
Primary atom site location: structure-invariant direct methods	Extinction coefficient: 0.0070 (10)

### Special details

**Table:**

Geometry. All e.s.d.'s (except the e.s.d. in the dihedral angle between two l.s. planes) are estimated using the full covariance matrix. The cell e.s.d.'s are taken into account individually in the estimation of e.s.d.'s in distances, angles and torsion angles; correlations between e.s.d.'s in cell parameters are only used when they are defined by crystal symmetry. An approximate (isotropic) treatment of cell e.s.d.'s is used for estimating e.s.d.'s involving l.s. planes.
Refinement. Refinement of *F*^2^ against ALL reflections. The weighted *R*-factor *wR* and goodness of fit *S* are based on *F*^2^, conventional *R*-factors *R* are based on *F*, with *F* set to zero for negative *F*^2^. The threshold expression of *F*^2^ > σ(*F*^2^) is used only for calculating *R*-factors(gt) *etc*. and is not relevant to the choice of reflections for refinement. *R*-factors based on *F*^2^ are statistically about twice as large as those based on *F*, and *R*- factors based on ALL data will be even larger.

### Fractional atomic coordinates and isotropic or equivalent isotropic displacement parameters (Å^2^)

**Table:**

	*x*	*y*	*z*	*U*_iso_*/*U*_eq_	
O1	0.48425 (14)	0.87273 (11)	0.09194 (5)	0.0573 (3)	
O2	0.67296 (15)	0.85457 (12)	0.04518 (6)	0.0658 (4)	
O3	0.22158 (12)	0.85200 (11)	−0.07008 (5)	0.0544 (3)	
O4	0.43549 (15)	0.53003 (11)	−0.09744 (6)	0.0657 (4)	
O5	0.4359 (2)	0.65360 (13)	−0.21784 (6)	0.0918 (6)	
O6	0.51056 (15)	0.84702 (11)	−0.20115 (6)	0.0647 (4)	
C1	0.1064 (2)	0.89144 (18)	0.03761 (9)	0.0603 (5)	
H1	0.0445	0.8860	0.0014	0.072*	
C2	0.0543 (2)	0.9138 (2)	0.09163 (10)	0.0710 (6)	
H2	−0.0425	0.9239	0.0922	0.085*	
C3	0.1467 (3)	0.9215 (2)	0.14565 (10)	0.0733 (6)	
H3	0.1113	0.9371	0.1824	0.088*	
C4	0.2887 (2)	0.90663 (18)	0.14566 (8)	0.0653 (5)	
H4	0.3500	0.9114	0.1821	0.078*	
C5	0.3403 (2)	0.88433 (15)	0.09076 (8)	0.0511 (4)	
C6	0.25104 (19)	0.87658 (14)	0.03612 (7)	0.0491 (4)	
C7	0.31477 (18)	0.85169 (14)	−0.01846 (7)	0.0468 (4)	
C8	0.45453 (18)	0.82799 (14)	−0.01720 (7)	0.0454 (4)	
C9	0.5464 (2)	0.85058 (15)	0.03936 (8)	0.0507 (4)	
C10	0.51915 (18)	0.78861 (15)	−0.07384 (7)	0.0455 (4)	
C11	0.28495 (19)	0.85679 (16)	−0.12630 (8)	0.0494 (4)	
C12	0.39806 (18)	0.75276 (14)	−0.12394 (7)	0.0464 (4)	
C13	0.3355 (2)	0.62767 (16)	−0.10734 (9)	0.0540 (5)	
C14	0.5728 (2)	0.55895 (16)	−0.07821 (8)	0.0541 (4)	
C15	0.62175 (19)	0.67960 (15)	−0.06576 (8)	0.0503 (4)	
C16	0.7656 (2)	0.6973 (2)	−0.05198 (9)	0.0659 (5)	
H16	0.8007	0.7778	−0.0451	0.079*	
C17	0.8591 (2)	0.5977 (2)	−0.04821 (10)	0.0802 (7)	
H17	0.9559	0.6109	−0.0386	0.096*	
C18	0.8067 (3)	0.4787 (2)	−0.05896 (10)	0.0804 (7)	
H18	0.8685	0.4111	−0.0558	0.097*	
H10	0.5703 (17)	0.8589 (16)	−0.0883 (7)	0.045 (4)*	
H11	0.3340 (17)	0.9362 (16)	−0.1275 (7)	0.047 (4)*	
H13A	0.268 (2)	0.5981 (18)	−0.1418 (9)	0.064 (5)*	
H13B	0.291 (2)	0.6358 (19)	−0.0683 (10)	0.071 (6)*	
C19	0.6654 (3)	0.45911 (19)	−0.07413 (9)	0.0686 (6)	
H19	0.6311	0.3786	−0.0817	0.082*	
C20	0.4500 (2)	0.74304 (16)	−0.18581 (8)	0.0537 (4)	
C21	0.5548 (3)	0.8502 (2)	−0.26125 (11)	0.0918 (8)	
H21A	0.5970	0.9297	−0.2676	0.138*	
H21B	0.4742	0.8378	−0.2917	0.138*	
H21C	0.6227	0.7852	−0.2646	0.138*	
C22	0.16930 (19)	0.85119 (15)	−0.17946 (8)	0.0520 (4)	
C23	0.0536 (2)	0.77419 (19)	−0.18095 (9)	0.0651 (5)	
H23	0.0416	0.7260	−0.1468	0.078*	
C24	−0.0450 (2)	0.7678 (2)	−0.23264 (10)	0.0728 (6)	
H24	−0.1227	0.7151	−0.2326	0.087*	
C25	−0.0313 (2)	0.8371 (2)	−0.28415 (9)	0.0672 (6)	
C26	0.0823 (2)	0.9161 (2)	−0.28170 (9)	0.0721 (6)	
H26	0.0928	0.9656	−0.3156	0.087*	
C27	0.1816 (2)	0.92392 (19)	−0.23030 (9)	0.0642 (5)	
H27	0.2574	0.9787	−0.2299	0.077*	
C28	−0.1372 (3)	0.8274 (3)	−0.34122 (10)	0.0941 (8)	
H28A	−0.2096	0.7686	−0.3345	0.141*	
H28B	−0.0901	0.7998	−0.3749	0.141*	
H28C	−0.1793	0.9077	−0.3506	0.141*	

### Atomic displacement parameters (Å^2^)

**Table:**

	*U*^11^	*U*^22^	*U*^33^	*U*^12^	*U*^13^	*U*^23^
O1	0.0720 (9)	0.0549 (7)	0.0397 (7)	−0.0028 (6)	−0.0132 (6)	−0.0050 (5)
O2	0.0612 (9)	0.0693 (9)	0.0601 (8)	−0.0020 (7)	−0.0184 (6)	−0.0081 (6)
O3	0.0575 (7)	0.0596 (7)	0.0412 (7)	0.0072 (6)	−0.0121 (5)	−0.0049 (5)
O4	0.0763 (9)	0.0365 (6)	0.0798 (9)	−0.0020 (6)	−0.0069 (7)	0.0053 (6)
O5	0.1751 (17)	0.0535 (8)	0.0456 (8)	−0.0130 (9)	0.0101 (9)	−0.0111 (6)
O6	0.0880 (10)	0.0519 (7)	0.0548 (8)	−0.0085 (6)	0.0122 (7)	−0.0012 (6)
C1	0.0694 (13)	0.0548 (10)	0.0540 (11)	0.0050 (9)	−0.0023 (9)	0.0028 (8)
C2	0.0788 (14)	0.0683 (13)	0.0671 (14)	0.0075 (11)	0.0134 (11)	0.0081 (10)
C3	0.0958 (18)	0.0695 (13)	0.0567 (13)	0.0042 (12)	0.0178 (12)	0.0050 (10)
C4	0.0954 (16)	0.0571 (11)	0.0405 (10)	−0.0036 (10)	−0.0023 (10)	0.0018 (8)
C5	0.0676 (12)	0.0365 (8)	0.0457 (10)	−0.0007 (7)	−0.0058 (8)	0.0022 (7)
C6	0.0665 (12)	0.0345 (8)	0.0431 (9)	0.0025 (7)	−0.0050 (8)	0.0012 (7)
C7	0.0594 (11)	0.0349 (8)	0.0412 (9)	0.0007 (7)	−0.0126 (7)	−0.0017 (6)
C8	0.0588 (11)	0.0330 (8)	0.0399 (9)	−0.0007 (7)	−0.0102 (7)	−0.0010 (6)
C9	0.0630 (12)	0.0366 (8)	0.0469 (10)	−0.0014 (8)	−0.0142 (8)	−0.0018 (7)
C10	0.0539 (10)	0.0362 (8)	0.0425 (9)	−0.0029 (7)	−0.0086 (7)	0.0007 (7)
C11	0.0598 (11)	0.0411 (9)	0.0430 (10)	0.0002 (8)	−0.0106 (8)	−0.0016 (7)
C12	0.0576 (10)	0.0360 (8)	0.0412 (9)	−0.0012 (7)	−0.0100 (7)	−0.0012 (7)
C13	0.0647 (12)	0.0393 (9)	0.0538 (11)	−0.0053 (8)	−0.0084 (9)	−0.0004 (8)
C14	0.0725 (13)	0.0435 (9)	0.0442 (10)	0.0049 (8)	−0.0008 (8)	0.0067 (7)
C15	0.0607 (11)	0.0461 (9)	0.0410 (9)	0.0062 (8)	−0.0054 (8)	0.0020 (7)
C16	0.0631 (12)	0.0650 (12)	0.0650 (13)	0.0057 (10)	−0.0097 (10)	−0.0008 (10)
C17	0.0667 (14)	0.0942 (18)	0.0759 (15)	0.0222 (12)	−0.0054 (11)	0.0034 (13)
C18	0.0985 (19)	0.0750 (15)	0.0663 (14)	0.0377 (13)	0.0046 (12)	0.0058 (11)
C19	0.0949 (17)	0.0496 (10)	0.0591 (12)	0.0166 (10)	0.0015 (11)	0.0074 (9)
C20	0.0721 (12)	0.0403 (9)	0.0441 (10)	0.0045 (8)	−0.0102 (8)	−0.0002 (7)
C21	0.137 (2)	0.0781 (16)	0.0653 (15)	−0.0017 (14)	0.0319 (14)	0.0077 (11)
C22	0.0636 (11)	0.0445 (9)	0.0426 (9)	0.0068 (8)	−0.0136 (8)	−0.0022 (7)
C23	0.0680 (12)	0.0648 (12)	0.0562 (12)	−0.0028 (10)	−0.0167 (9)	0.0075 (9)
C24	0.0696 (13)	0.0672 (13)	0.0726 (14)	0.0010 (10)	−0.0258 (11)	−0.0026 (11)
C25	0.0730 (13)	0.0730 (13)	0.0491 (11)	0.0267 (11)	−0.0169 (9)	−0.0156 (10)
C26	0.0854 (15)	0.0824 (14)	0.0443 (11)	0.0201 (12)	−0.0078 (10)	0.0087 (10)
C27	0.0725 (13)	0.0611 (11)	0.0541 (11)	0.0063 (9)	−0.0100 (9)	0.0081 (9)
C28	0.0876 (16)	0.120 (2)	0.0632 (14)	0.0365 (15)	−0.0328 (12)	−0.0224 (13)

### Geometric parameters (Å, º)

**Table:**

O1—C5	1.373 (2)	C12—C13	1.530 (2)
O1—C9	1.385 (2)	C13—H13A	0.98 (2)
O2—C9	1.197 (2)	C13—H13B	1.01 (2)
O3—C7	1.3473 (19)	C14—C19	1.382 (3)
O3—C11	1.444 (2)	C14—C15	1.389 (2)
O4—C14	1.358 (2)	C15—C16	1.378 (3)
O4—C13	1.412 (2)	C16—C17	1.386 (3)
O5—C20	1.186 (2)	C16—H16	0.9300
O6—C20	1.318 (2)	C17—C18	1.378 (3)
O6—C21	1.437 (2)	C17—H17	0.9300
C1—C2	1.366 (3)	C18—C19	1.360 (3)
C1—C6	1.392 (3)	C18—H18	0.9300
C1—H1	0.9300	C19—H19	0.9300
C2—C3	1.387 (3)	C21—H21A	0.9600
C2—H2	0.9300	C21—H21B	0.9600
C3—C4	1.362 (3)	C21—H21C	0.9600
C3—H3	0.9300	C22—C23	1.374 (3)
C4—C5	1.381 (3)	C22—C27	1.379 (3)
C4—H4	0.9300	C23—C24	1.379 (3)
C5—C6	1.382 (2)	C23—H23	0.9300
C6—C7	1.437 (2)	C24—C25	1.374 (3)
C7—C8	1.352 (2)	C24—H24	0.9300
C8—C9	1.445 (2)	C25—C26	1.369 (3)
C8—C10	1.518 (2)	C25—C28	1.508 (3)
C10—C15	1.518 (2)	C26—C27	1.379 (3)
C10—C12	1.537 (2)	C26—H26	0.9300
C10—H10	0.972 (17)	C27—H27	0.9300
C11—C22	1.500 (2)	C28—H28A	0.9600
C11—C12	1.546 (2)	C28—H28B	0.9600
C11—H11	0.973 (17)	C28—H28C	0.9600
C12—C20	1.509 (3)		
			
C5—O1—C9	122.17 (13)	C12—C13—H13B	110.3 (12)
C7—O3—C11	114.68 (13)	H13A—C13—H13B	112.1 (16)
C14—O4—C13	118.86 (13)	O4—C14—C19	115.14 (17)
C20—O6—C21	116.42 (16)	O4—C14—C15	123.98 (15)
C2—C1—C6	120.83 (19)	C19—C14—C15	120.83 (19)
C2—C1—H1	119.6	C16—C15—C14	118.07 (16)
C6—C1—H1	119.6	C16—C15—C10	121.77 (16)
C1—C2—C3	119.5 (2)	C14—C15—C10	119.87 (16)
C1—C2—H2	120.2	C15—C16—C17	121.3 (2)
C3—C2—H2	120.2	C15—C16—H16	119.3
C4—C3—C2	121.0 (2)	C17—C16—H16	119.3
C4—C3—H3	119.5	C18—C17—C16	119.1 (2)
C2—C3—H3	119.5	C18—C17—H17	120.4
C3—C4—C5	119.05 (19)	C16—C17—H17	120.4
C3—C4—H4	120.5	C19—C18—C17	120.7 (2)
C5—C4—H4	120.5	C19—C18—H18	119.7
O1—C5—C4	117.78 (16)	C17—C18—H18	119.7
O1—C5—C6	120.81 (16)	C18—C19—C14	120.0 (2)
C4—C5—C6	121.39 (19)	C18—C19—H19	120.0
C5—C6—C1	118.25 (17)	C14—C19—H19	120.0
C5—C6—C7	117.22 (17)	O5—C20—O6	123.41 (18)
C1—C6—C7	124.53 (16)	O5—C20—C12	124.40 (17)
O3—C7—C8	123.92 (16)	O6—C20—C12	112.17 (14)
O3—C7—C6	113.50 (15)	O6—C21—H21A	109.5
C8—C7—C6	122.57 (15)	O6—C21—H21B	109.5
C7—C8—C9	118.14 (16)	H21A—C21—H21B	109.5
C7—C8—C10	122.53 (14)	O6—C21—H21C	109.5
C9—C8—C10	119.16 (15)	H21A—C21—H21C	109.5
O2—C9—O1	115.95 (15)	H21B—C21—H21C	109.5
O2—C9—C8	126.01 (18)	C23—C22—C27	118.11 (16)
O1—C9—C8	118.02 (16)	C23—C22—C11	123.31 (16)
C8—C10—C15	116.16 (13)	C27—C22—C11	118.55 (17)
C8—C10—C12	108.09 (14)	C22—C23—C24	120.59 (19)
C15—C10—C12	107.70 (13)	C22—C23—H23	119.7
C8—C10—H10	109.0 (9)	C24—C23—H23	119.7
C15—C10—H10	106.9 (9)	C25—C24—C23	121.6 (2)
C12—C10—H10	108.7 (10)	C25—C24—H24	119.2
O3—C11—C22	108.59 (15)	C23—C24—H24	119.2
O3—C11—C12	108.18 (13)	C26—C25—C24	117.38 (18)
C22—C11—C12	115.84 (14)	C26—C25—C28	121.1 (2)
O3—C11—H11	108.1 (10)	C24—C25—C28	121.6 (2)
C22—C11—H11	108.7 (10)	C25—C26—C27	121.7 (2)
C12—C11—H11	107.2 (10)	C25—C26—H26	119.2
C20—C12—C13	109.72 (14)	C27—C26—H26	119.2
C20—C12—C10	111.07 (15)	C26—C27—C22	120.6 (2)
C13—C12—C10	109.11 (13)	C26—C27—H27	119.7
C20—C12—C11	109.12 (13)	C22—C27—H27	119.7
C13—C12—C11	110.36 (15)	C25—C28—H28A	109.5
C10—C12—C11	107.42 (13)	C25—C28—H28B	109.5
O4—C13—C12	114.18 (16)	H28A—C28—H28B	109.5
O4—C13—H13A	104.0 (11)	C25—C28—H28C	109.5
C12—C13—H13A	109.6 (11)	H28A—C28—H28C	109.5
O4—C13—H13B	106.5 (12)	H28B—C28—H28C	109.5
			
C6—C1—C2—C3	−0.2 (3)	O3—C11—C12—C10	−68.00 (17)
C1—C2—C3—C4	−0.2 (3)	C22—C11—C12—C10	169.86 (15)
C2—C3—C4—C5	0.4 (3)	C14—O4—C13—C12	26.9 (2)
C9—O1—C5—C4	−179.86 (15)	C20—C12—C13—O4	66.43 (19)
C9—O1—C5—C6	−1.3 (2)	C10—C12—C13—O4	−55.5 (2)
C3—C4—C5—O1	178.43 (17)	C11—C12—C13—O4	−173.29 (14)
C3—C4—C5—C6	−0.2 (3)	C13—O4—C14—C19	−175.30 (17)
O1—C5—C6—C1	−178.79 (15)	C13—O4—C14—C15	1.9 (3)
C4—C5—C6—C1	−0.2 (2)	O4—C14—C15—C16	−174.18 (17)
O1—C5—C6—C7	2.1 (2)	C19—C14—C15—C16	2.9 (3)
C4—C5—C6—C7	−179.32 (16)	O4—C14—C15—C10	−0.3 (3)
C2—C1—C6—C5	0.4 (3)	C19—C14—C15—C10	176.75 (16)
C2—C1—C6—C7	179.41 (17)	C8—C10—C15—C16	−93.5 (2)
C11—O3—C7—C8	−15.2 (2)	C12—C10—C15—C16	145.19 (17)
C11—O3—C7—C6	166.09 (13)	C8—C10—C15—C14	92.91 (19)
C5—C6—C7—O3	−176.76 (13)	C12—C10—C15—C14	−28.4 (2)
C1—C6—C7—O3	4.2 (2)	C14—C15—C16—C17	−2.5 (3)
C5—C6—C7—C8	4.5 (2)	C10—C15—C16—C17	−176.20 (18)
C1—C6—C7—C8	−174.56 (16)	C15—C16—C17—C18	0.5 (3)
O3—C7—C8—C9	170.02 (14)	C16—C17—C18—C19	1.1 (3)
C6—C7—C8—C9	−11.3 (2)	C17—C18—C19—C14	−0.7 (3)
O3—C7—C8—C10	−5.1 (2)	O4—C14—C19—C18	175.98 (18)
C6—C7—C8—C10	173.51 (14)	C15—C14—C19—C18	−1.4 (3)
C5—O1—C9—O2	172.88 (14)	C21—O6—C20—O5	2.7 (3)
C5—O1—C9—C8	−5.7 (2)	C21—O6—C20—C12	−175.78 (17)
C7—C8—C9—O2	−166.65 (16)	C13—C12—C20—O5	4.2 (3)
C10—C8—C9—O2	8.7 (2)	C10—C12—C20—O5	124.9 (2)
C7—C8—C9—O1	11.8 (2)	C11—C12—C20—O5	−116.8 (2)
C10—C8—C9—O1	−172.92 (13)	C13—C12—C20—O6	−177.29 (14)
C7—C8—C10—C15	−134.14 (16)	C10—C12—C20—O6	−56.57 (18)
C9—C8—C10—C15	50.8 (2)	C11—C12—C20—O6	61.68 (19)
C7—C8—C10—C12	−13.0 (2)	O3—C11—C22—C23	−40.9 (2)
C9—C8—C10—C12	171.88 (13)	C12—C11—C22—C23	81.0 (2)
C7—O3—C11—C22	177.67 (13)	O3—C11—C22—C27	141.21 (17)
C7—O3—C11—C12	51.19 (17)	C12—C11—C22—C27	−96.9 (2)
C8—C10—C12—C20	166.11 (12)	C27—C22—C23—C24	1.8 (3)
C15—C10—C12—C20	−67.66 (17)	C11—C22—C23—C24	−176.10 (18)
C8—C10—C12—C13	−72.81 (17)	C22—C23—C24—C25	0.1 (3)
C15—C10—C12—C13	53.43 (19)	C23—C24—C25—C26	−1.8 (3)
C8—C10—C12—C11	46.84 (17)	C23—C24—C25—C28	178.4 (2)
C15—C10—C12—C11	173.07 (14)	C24—C25—C26—C27	1.6 (3)
O3—C11—C12—C20	171.49 (13)	C28—C25—C26—C27	−178.60 (19)
C22—C11—C12—C20	49.4 (2)	C25—C26—C27—C22	0.3 (3)
O3—C11—C12—C13	50.85 (17)	C23—C22—C27—C26	−2.0 (3)
C22—C11—C12—C13	−71.3 (2)	C11—C22—C27—C26	175.98 (18)

### Hydrogen-bond geometry (Å, º)

**Table:**

*D*—H···*A*	*D*—H	H···*A*	*D*···*A*	*D*—H···*A*
C4—H4···O5^i^	0.93	2.35	3.209 (2)	153
C21—H21*A*···O5^ii^	0.96	2.43	3.285 (3)	148

Symmetry codes: (i) *x*, −*y*+3/2, *z*+1/2; (ii) −*x*+1, *y*+1/2, −*z*−1/2.
